# Sentinel lymph nodes in melanoma: necessary as ever for optimal treatment

**DOI:** 10.1007/s10585-023-10254-2

**Published:** 2024-01-02

**Authors:** Mark B. Faries

**Affiliations:** The Angeles Clinic and Research Institute, a Cedars-Sinai Affiliate, Los Angeles, CA USA

**Keywords:** Melanoma, Sentinel lymph node, Biomarker, De-escalation

## Abstract

Lymphatic metastasis is the dominant route of initial spread for most solid tumors. For many such malignancies, including melanomas, surgical treatment previously included removal of all potentially draining regional lymph nodes (elective node dissection). The advent of lymphatic mapping and sentinel lymph node (SLN) biopsy allowed accurate pathologic assessment of the metastatic status of regional nodes and spared patients full dissection if their SLN was clear. In melanoma, recent clinical research has demonstrated that complete lymph node dissection is not clinically beneficial, even for patients with sentinel node metastases and that patients with high-risk primary melanomas benefit from adjuvant systemic immunotherapy, even without nodal disease. These two changes in the standard of care have led to some interest in abandoning surgical nodal staging via the sentinel lymph node biopsy procedure. However, this appears to be premature and potentially detrimental to optimal patient management. The ongoing value of sentinel node biopsy stems from its ability to provide critically important prognostic information as well as durable regional nodal disease control for most patients with nodal metastases, even in the absence of complete dissection of the basin. It also provides an opportunity to identify novel prognostic and predictive immunologic and molecular biomarkers. While it is certainly possible that additional changes in melanoma therapy will make sentinel lymph node biopsy obsolete in the future, at present it remains a minimally invasive, low morbidity means of improving both staging and outcomes.

## Introduction

The vast majority of solid tumors tend to metastasize initially via lymphatic channels to regional lymph nodes. In many cancer types there has been a longstanding controversy regarding the implications of this pattern of spread, particularly with regard to the utility of regional nodal surgery at the time of disease presentation, even in the absence of clinically apparent nodal metastases. Melanoma is a very early example of this debate. Herbert Snow, a London surgeon, is credited with initiating the debate through his report in the Lance in 1892, “Melanotic Cancerous Disease” in which he proposed “anticipatory gland excision.” [1] That therapeutic strategy would later be known as elective lymph node dissection (ELND) and consisted of complete removal of nodes and lymphatic tissue from the regional nodal basin(s) that were presumed to be the initial site at risk for dissemination. Snow noted that this spread often occurred before the skin primary site had progressed substantially stating, “…the apparent triviality of the pigmented papilloma which precedes melanocytic growths on the skin commonly fails to excite suspicion of danger until there is extensive implication of the lymph glands…” He noted that waiting until the regional disease became detectable on exam was ill advised,

We thus seen the utter futility of operative measures which are addressed to the primary lesion only… We further see the paramount importance of securing, whenever possible, the perfect eradication of those lymph glands which will necessarily be first infected; before enlargement takes place radical removal of such organs in the axilla, groin, surface regions of the neck &c, before they have undergone appreciable increase in bulk, is a safe and easy measure, which, under the conditions indicated, should never be neglected.

However, this approach is not without costs. Complete dissection of nodal basins is attended by a real risk for both short and long-term morbidity [2]. These risks vary somewhat by which basin is involved, but include infection, seroma, wound healing problems and nerve injury in the short term and lymphedema over the long term. As melanoma was increasingly diagnosed at earlier stages, most patients were found to have only normal lymph nodes at the time of dissection. This led many to question the wisdom of ELND (see Table 1).

**Table 1:** Pro and Con Arguments for Sentinel Lymph Node Biopsy in Melanoma

	Pro	Con	Comments
**Staging and Prognosis**	Among the strongest prognostic factors	Some node-negative patients still recur	Gene expression profiling appears to add to, rather than replace pathologic staging
**Melanoma-specific survival**	Evidence for substantial survival benefit for node-positive, thin - intermediate thickness melanomas	No MSS benefit for entire population (node negative cannot benefit)	
**Disease control**	Marked (~ 80%) reduction in regional recurrence; no longer requires complete dissection to achieve	Many nodal recurrences can be salvaged, but currently requires complete dissection	Recent neoadjuvant data suggest surgery for recurrence may be de-escalated in some
**Risk Stratification**	Accurate staging makes optimal stratification for clinical trials	Easier trial accrual at centers opposed to sentinel node biopsy	
**Morbidity**	Complications uncommon and generally short-lived	Possible toxicity even with a minimally invasive operation	
**Cost**	Markedly less than that of current adjuvant therapies	More costly than wide excision alone	
**Innovation**	Immune profiling sentinel nodes examines initial tumor-host interaction	Blood or tumor tissue also possible sites for translational research	

Multiple randomized clinical trials were completed examining the role of ELND in melanoma [3–7]. None of these showed a statistically significant survival benefit of dissection, but there was enough of a trend in that direction for many of the studies, as well as substantial subgroups with apparent benefit that the technique remained of interest to many. As is often the case, a different therapeutic advance altered the therapeutic landscape to such an extent that the debate surrounding ELND became moot. That advance was the development of lymphatic mapping and sentinel lymph node (SLN) biopsy by Morton and colleagues [8]. This technique allowed identification of the specific lymph node (or small number of lymph nodes) that receive direct lymphatic drainage from the primary tumor site. The pathologic status of the SLN proved an accurate representation of the overall nodal basin, and those patients whose SLNs were clear could be spared the larger, more morbid operation.

At the time of its initial development, SLN biopsy was only intended to be a means of selecting patients for complete lymph node dissection [9]. This second operation was a standard recommendation for any patient with SLN metastasis. However, it became apparent over time that the vast majority of patients with SLN disease did not harbor additional metastases in the remainder of their lymph nodes, leading randomized trials of so-called completion lymph node dissection, discussed below [10].

SLN biopsy became a standard recommendation for appropriately selected patients with melanoma in all major national and professional guidelines [11–14]. The value at the time was for staging and thereby selection for adjuvant therapy. Initially the only option for medical therapy was interferon, which was toxic and had limited therapeutic value, making accurate prognostic assessment critical [15]. More recently, both targeted and immune checkpoint therapies have been developed and approved, making adjuvant therapy more broadly appealing [16, 17]. For patients with clinically localized disease, the only means of accessing these treatments was through identification of occult nodal metastases using SLN biopsy. As noted below, the ground has again shifted, changing these rationales and leading to another debate on SLN utility.

## New arguments for de-escalation and omitting SLN biopsy

Two large, prospective clinical trials were recently completed evaluating the value of completion lymph node dissection for melanoma patients with SLN metastases. These trials performed by the German Dermato-oncology Cooperative Group (DeCOG-SLT) and the Multicenter Selective Lymphadenectomy Group (MSLT-II) randomized patients who had been found to have SLN metastases to either immediate completion lymph node dissection or nodal observation with ultrasound [18, 19]. Both trials showed no discernable effect of dissection on melanoma-specific or overall survival, though there appeared to be some benefit in regional nodal recurrence and provision of some prognostic information. These observations led to a change in the standard of care and acceptance of nodal observation as a means of managing these patients.

A second, even more recent development occurred though two randomized clinical trials evaluating Programmed Death-1 (PD-1) blockade immunotherapy in the adjuvant setting for Stage IIB/C patients [20, 21]. Both trials confirmed a significant reduction in the risk of relapse associated with treatment, making these patients candidates for standard adjuvant therapy. Since nodal metastases were not strictly required for patients to qualify for systemic therapy, some began to call for omission of SLN biopsy for patients with clinical Stage IIB/C melanoma. In addition, these trials have only documented benefits in recurrence-free survival, not overall survival. The data to date do not show a reduction in nodal recurrence with systemic therapy, and currently all patients with nodal recurrences would undergo complete nodal dissections rather than the less morbid SLN biopsy. The most appropriate management of these patients, who have recurred despite PD-1 blockade is also uncertain at this time.

## Reasons for retaining SLN biopsy

These calls, however, may be premature. At present, there is significant remaining value that patients derive from the sentinel node procedure. These benefits stem from the value of the prognostic/staging information derived from the pathologic evaluation of the SLN and from the therapeutic effect of removal of nodal metastasis at an early stage.

In terms of staging, there are few pieces of information that carry more value in determining a patient’s prognosis than the presence or absence of nodal metastases [22]. While it is true that some patients with Stage II melanoma have worse outcomes than some with Stage III, comparison of prognosis between a patient with a negative SLN and one with the same tumor, but a SLN metastasis, always shows a marked decrease in survival for the patient with metastasis [23]. The extent to which the predicted survival decreases varies based on the overall stage and the volume of disease found in the SLN.

Why does an accurate assessment of prognosis matter? First, many patients find this information important, as it affects their plans for treatment, follow up and life in general. Most specifically, deciding whether to pursue adjuvant medical therapy. While the options for adjuvant medical therapy have improved dramatically with the development of both targeted and immune checkpoint blockade drugs, these therapies are not without risk and cost. Different patients will have different prognostic thresholds for electing adjuvant therapy but having the most accurate assessment of the risk of recurrence and death is essential to making the best decision. For example, a patient with Stage IIB melanoma would be expected to have a 5-year melanoma-specific survival of 87%, but presence of a SLN metastasis would reduce that expectation to 69% [22]. Many patients would reasonably decide not to undergo one year of PD-1 blockade with the former situation, but the latter would be much more likely to feel the risks of treatment were justified. Similar considerations apply to recommendations for clinical monitoring. For example, a patient with a T2a primary melanoma would need relatively limited follow up and no imaging if their SLN was free of disease but would need more thorough follow up with at least nodal basin ultrasound if their SLN contained melanoma. With a greater disease burden in the SLN, the same patient might elect adjuvant therapy.

For clinicians and clinical researchers, SLN staging also remains important. Being able to accurately compare outcomes among groups of patients requires accurate staging information. This need is demonstrated well by studies that pre-date the SLN era in which patients who were only staged clinically, or even with elective lymph node dissection as having negative nodes had surprisingly poor outcomes. The degree of uncertainty in prognosis makes interpreting any clinical series or trial very difficult and nearly impossible to compare to any other report. In clinical trial design, this is also an important consideration. Accurate stratification of any randomized trial depends on accurate prognostication. Although there are now trials that have opened that do not require SLN staging for patients with clinical Stage IIB/C melanoma, there is a legitimate concern that interpretation of those trials will be difficult due to the lack of accurate staging. An example of this comes from a randomized trial that examined margin width for primary melanoma excisions [24]. That trial compared a 1 cm margin to a 3 cm margin and reported improved outcomes for patients with the wider margin. However, SLN biopsy was not used in that trial and the principal difference in outcome was in nodal recurrence. This leads to speculation that the arms were imbalanced at the time of randomization and that imbalance, rather than any therapeutic effect of a wider margin led to the survival differences. As a result, that trial has largely fallen out of relevance today, and a similar fate could await new trials that prematurely abandon SLN staging.

Establishing and maintaining regional nodal disease control is also an important and often forgotten therapeutic goal. Macroscopic nodal disease requires more extensive surgery to clear, which leads to greater short and long-term morbidity [9]. Loss of regional disease control, which is more likely in the setting of macroscopic nodal metastases is a tragedy for patients, impairing both quality and likely duration of life. Use of SLN biopsy can reduce the frequency of clinical nodal recurrences, allowing many patients to avoid more extensive treatment such as complete lymph node dissection. It is important to recognize that obtaining long lasting regional disease control, even among patients with nodal metastases at presentation, requires only the minimally invasive SLN biopsy in most cases [19]. Evidence for this comes from the MSLT-II study in which half of the patients (all of whom had sentinel node metastases) were randomized to nodal observation and only had additional nodal surgery in the event of nodal recurrence. The vast majority (~ 80%) never had such a recurrence, indicating all of their regional nodal disease had been removed in the SLN biopsy [25]. Clearly some patients have an increased risk for non-SLN disease and therefore nodal recurrence. Analysis of the MSLT-II data identified increased Breslow thickness, older age, non-axillary nodal basin and SLN tumor burden as factors related to nodal recurrence and combining those factors into a score allows estimation of in-basin nodal recurrence risk. However, even in the highest risk group, a majority of patients never suffer nodal recurrence.

The SLN is also an invaluable resource for discovery that may improve outcomes of future generations of melanoma patients. The node, as the location of the first interaction between the immune system and metastatic melanoma, is a rich source of information about the character of that interaction. It is increasingly clear that the immune competence of the node is related to patient outcomes. One potential measure of that competence can be derived during the SLN procedure, which is dependent on active uptake of radioactive tracer by antigen presenting cells located in the node [26, 27]. This produces the radioactive counts that allow surgical identification of the node. Quantification of this uptake in the radioactive counts found in the node reveal interesting relationships to nodal function and patient outcome. Patients with high counts are more likely to harbor SLN metastases but have lower rates of in-transit and distant disease. Furthermore, they have improved melanoma specific survival. Loss of uptake seems to occur as lymph nodes age, which may help explain a long-standing paradox in melanoma in which nodal metastasis (a strong prognostic indicator) is less common in older patients, even though their outcomes are worse. The biologic source of this loss of function is not completely understood, but an association with decreased HAPLN-1 in older individuals (in both murine model and patients) results in loss of lymphatic vessel integrity and alteration in the patterns of metastasis [27]. Other important changes in nodal immune function are also likely to be involved and may yield additional therapeutic targets in the future.

## Future perspectives

The role of SLN biopsy in melanoma is likely to continue to evolve. Current selection of patients for SLN biopsy is based on clinical factors, with the National Comprehensive Cancer Network recommending biopsy be discussed and offered for patients with T2 melanoma and discussed and considered for those with T1b melanomas [28]. A more nuanced approach can be taken with the help of nomograms that use clinical data to estimate the risk of nodal involvement. The most recent and possibly best so far comes from the Melanoma Institute Australia and uses age, Breslow thickness, mitotic rate, ulceration, histologic subtype and lymphovascular invasion, which can be entered into an online resource which then performs the calculation of risk [29]. There has been some consensus that patients with a greater than 10% risk should be offered the procedure and those with a 5–10% risk should consider it. The advent of Gene Expression Profiling may allow refinement of these estimates, and several such tests have been developed [30]. Prospective validation of any added value to more readily available and cost-free clinical models are lacking as of now, but may come in the future.

The other major change in surgical management of melanoma, neoadjuvant therapy, may also become an area of change and evolution in SLN biopsy. There is growing, convincing evidence that for patients with clinically apparent nodal metastases, inserting a short course of PD-1 blockade prior to surgical resection markedly improves clinical outcomes and allows improved prognostication for patients based on the pathologic tumor response seen at surgery [31]. There are also ongoing clinical trials investigating neoadjuvant therapy in high-risk melanomas prior to sentinel node biopsy. In theory this change in timing may allow re-activation of immune cells in the SLN node that may not only eradicate the nodal disease, but also exit the node and boost systemic anti-melanoma immunity. These trials will be of great interest in the next few years.

As has been the case in the past, discoveries made in this way in melanoma may come to influence treatment in other cancers including breast cancer. For example, it appears immune response is critically important to outcomes and treatment in triple negative breast cancer [32]. Improved knowledge of immune and molecular features of disease progression or resistance in melanoma may inform breast cancer, just as checkpoint inhibition, which began in melanoma, has come to be an important resource in triple negative breast cancer. Similarly, responses to neoadjuvant immunotherapy prior to SLN biopsy in melanoma may lead to similar inroads in breast cancer and other malignancies.

## Conclusion

Melanoma treatment has advanced at an incredible pace in recent years, changing any number of paradigms. This is true in surgical therapy in the same way it has been in systemic treatments, and the role of SLN biopsy has evolved. However, the procedure retains an important place in the most effective and rational treatment of patients. This is due to its ability to risk stratify patients to help make informed treatment decisions and conduct balanced clinical trials. It also provides a therapeutic benefit in providing regional disease control for most patients with regional nodal disease without the need to resort to complete and potentially more morbid complete nodal dissection. The role of SLN biopsy will undoubtedly continue to evolve.
